# Giant right and left atrium: spectrum of rheumatic triple valve disease (double hit with double impact): a case report and review of the literature

**DOI:** 10.1186/s43044-024-00538-7

**Published:** 2024-08-13

**Authors:** Uma Devi Karuru, Sadanand Reddy Tummala, Paladugu Srinivas Gautam, T. Naveen, Kiran Kumar Kanjerla, Sai Kumar Mysore

**Affiliations:** Department of Cardiology, ESIC Medical College and Super Speciality Hospital, Hyderabad, India

**Keywords:** Giant right atrium, Giant left atrium, Rheumatic heart disease

## Abstract

**Background:**

Rheumatic heart disease (RHD) continues to pose a significant burden on global health, particularly in socioeconomically disadvantaged populations. We present the case of a 38-year-old woman with severe multivalvular RHD and giant atria, highlighting the challenges and complexities of managing this condition.

**Case presentation:**

The patient presented with progressively worsening dyspnoea, signs of right heart failure, and severe valvular abnormalities. Diagnostic evaluations revealed severe mixed mitral valve disease in the form of mitral stenosis and regurgitation, along with involvement of the aortic and tricuspid valves, leading to significant enlargement of both atria. Despite facing socioeconomic constraints and poor adherence to treatment, the patient underwent successful surgical intervention, resulting in remarkable symptomatic improvement.

**Conclusions:**

Through this case, we emphasise the importance of early detection, comprehensive management strategies, and multidisciplinary care in addressing the complexities of RHD. Despite the challenges posed by socioeconomic disparities, positive outcomes can be achieved with timely diagnosis and appropriate intervention. This case underscores the need for targeted efforts to improve access to healthcare resources and reduce the global burden of RHD.

**Supplementary Information:**

The online version contains supplementary material available at 10.1186/s43044-024-00538-7.

## Background

Rheumatic heart disease (RHD) presents a persistent challenge, especially in disadvantaged populations. Here, we detail the case of a 38-year-old woman with severe multivalvular RHD and giant atria. Despite socioeconomic constraints, she underwent successful surgical intervention. This case underscores the complexities of RHD management and highlights the importance of early detection and comprehensive care. Addressing socioeconomic barriers and advocating for improved healthcare access are crucial in alleviating the global burden of RHD.

## Case presentation

A 38-year-old woman, from a disadvantaged socioeconomic background, presented to our medical centre with a history of progressively worsening dyspnoea over the past 3 years. Initially categorised as class I, her symptoms worsened to class IV, sporadic haemoptysis, voice alterations accompanied by evident signs of right heart failure including anasarca, distended neck veins, fatigue, palpitations, and intermittent chest discomfort.

Upon examination, she was severely emaciated and displayed tachypnoea, tachycardia, markedly elevated jugular venous pressure, anasarca, and heart murmurs indicative of severe valve abnormalities: a grade V/VI pansystolic murmur in mitral area with radiation to the axilla and back, a short interval between the second heart sound (S2) to opening snap, and a grade IV mid-diastolic murmur without presystolic accentuation.

### Past history

She was diagnosed of RHD at the age of 20 years. She was recommended treatment involving medications and regular 3 weekly penicillin injections, though adherence was poor due to various constraints. She lost to follow-up.

### Family history

She had three children and hailed from a socioeconomically disadvantaged environment with limited access to clean water and suboptimal hygiene practices.

### Investigations

**Fig. 1:**
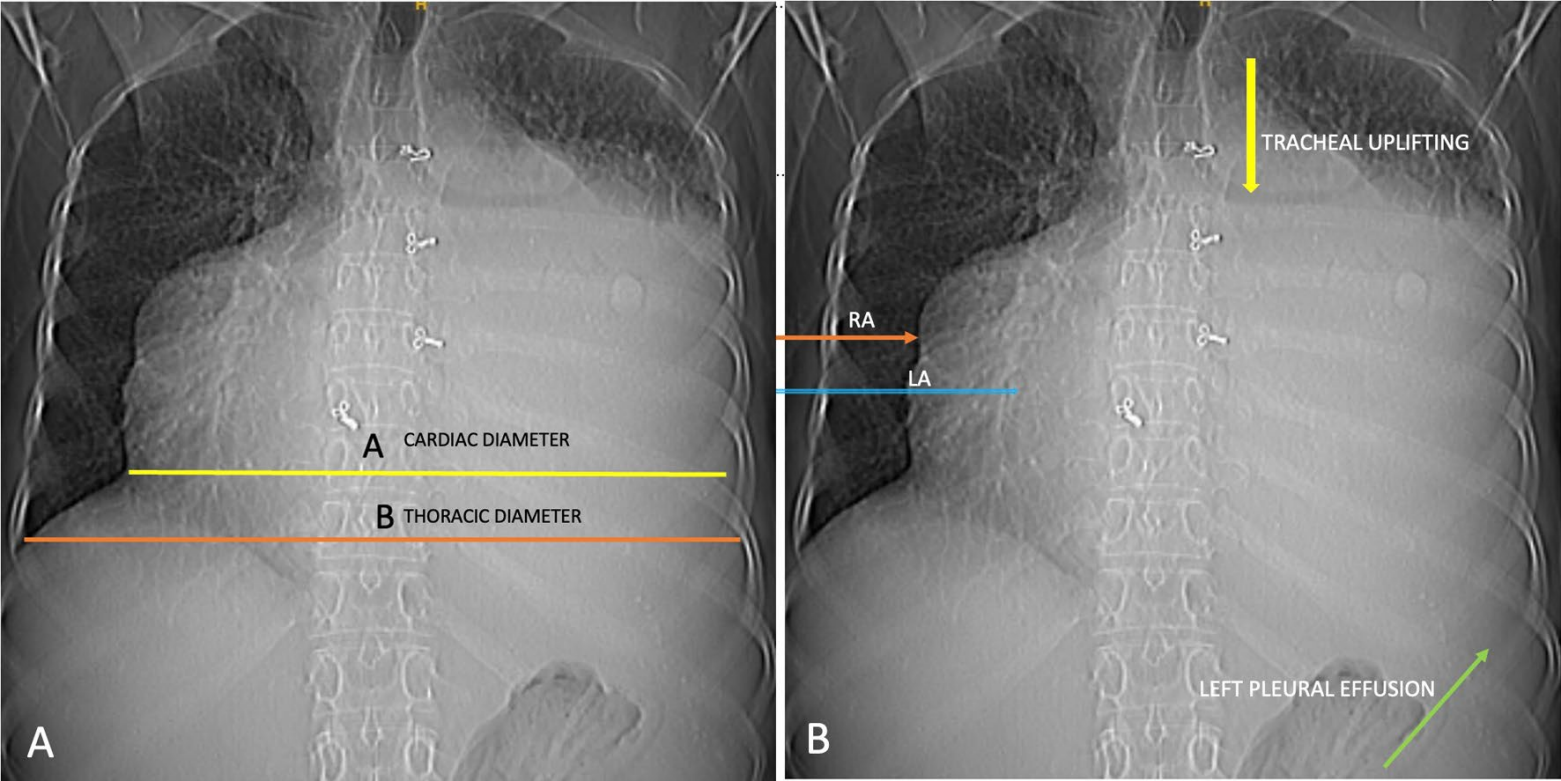
Chest X-ray. **A** Chest X-ray shows cardiothoracic ratio of 0.88 and **B** chest X-ray demonstrating dilated right atrium (RA), dilated left atrium (LA), tracheal uplifting due to LA enlargement, and left pleural effusion

**Fig. 2:**
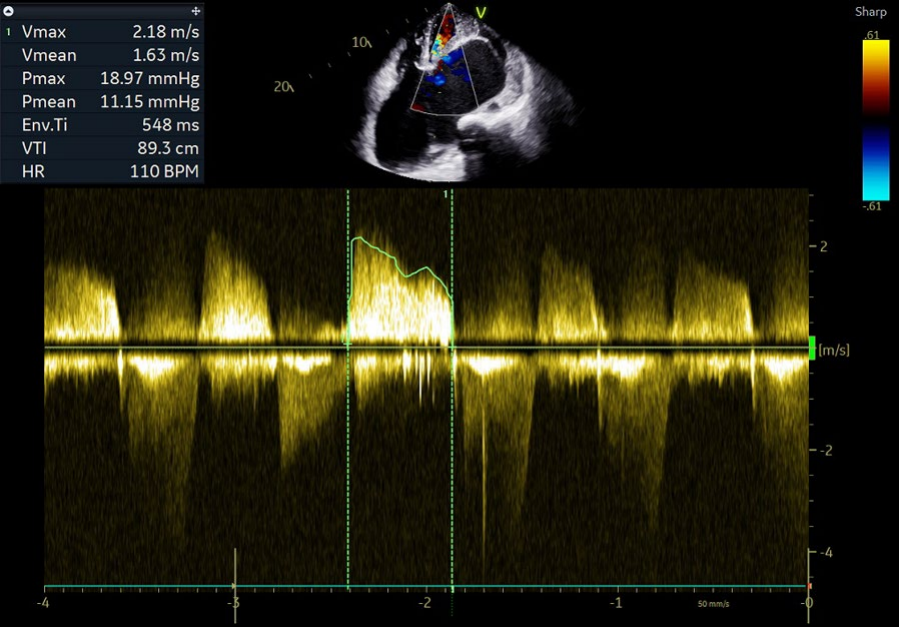
Two-dimensional echocardiographic assessment of severe mitral stenosis gradient and atrial fibrillation

**Fig. 3:**
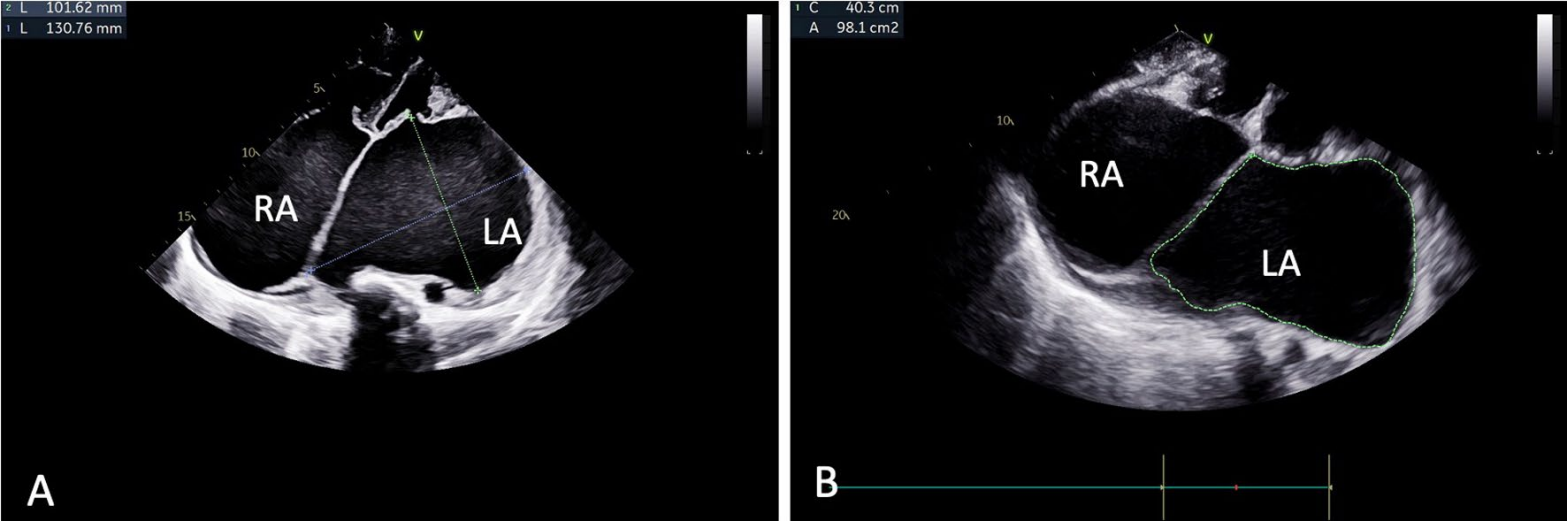
Two-dimensional echocardiography shows giant left atrium with dimensions of 13.0 × 10.1 cms (**A**) and area of 98.1 cm^2^ (**B**)

**Fig. 4:**
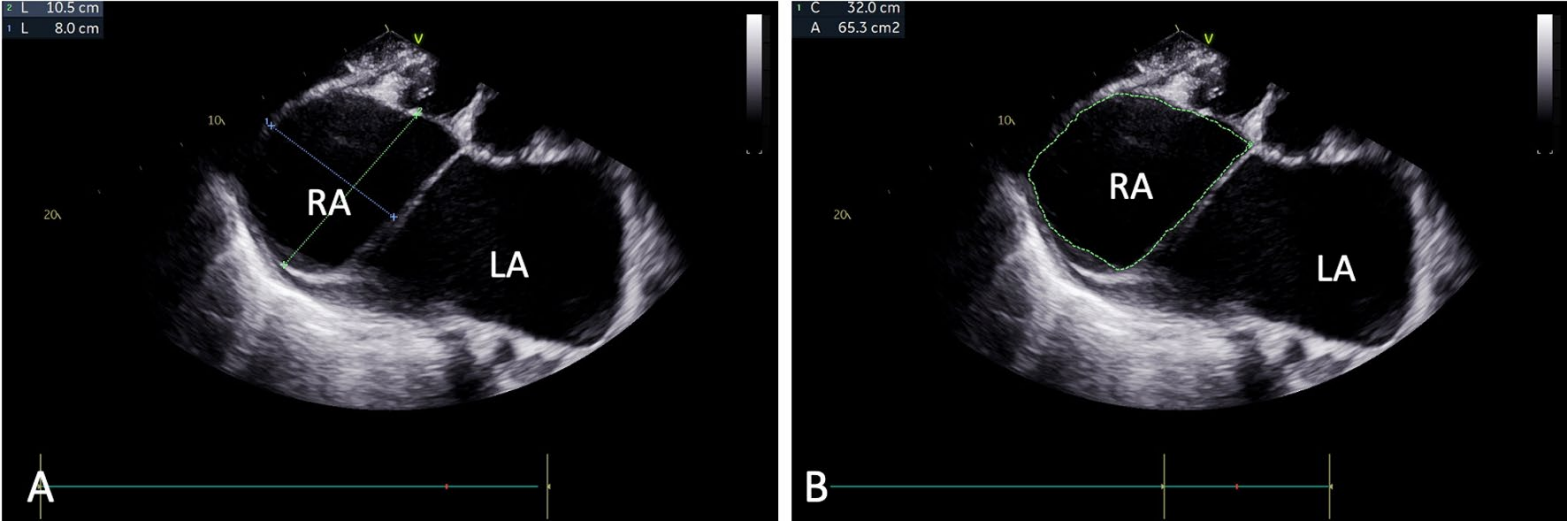
Two-dimensional echocardiography shows giant right atrium with dimensions of 10.5 × 8.0 cms (**A**) and area of 65.3 cm^2^ (**B**)

**Fig. 5:**
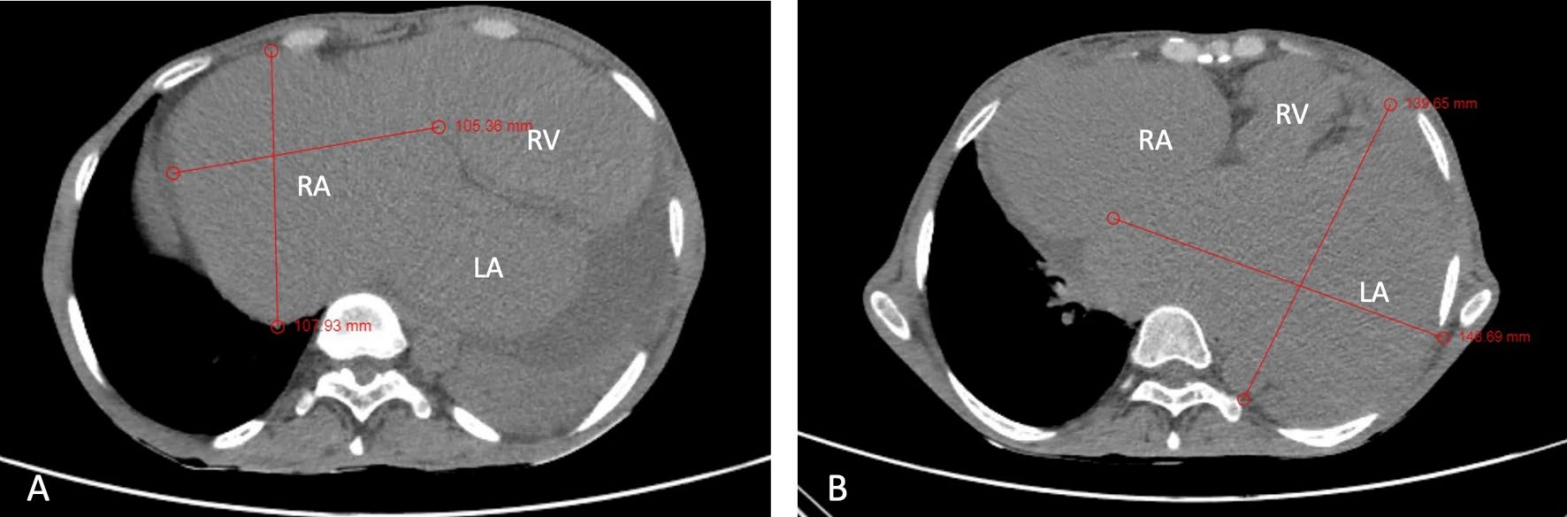
Cardiac computerised tomographic image of giant right atrium with dimensions of 10.7 × 10.5 cms (**A**) and giant left atrium with dimensions of 14.8 × 13.9 cms (**B**)

Electrocardiography (ECG) showed atrial fibrillation with a rapid ventricular response, while chest X-ray (CXR) exhibited significant cardiomegaly, enlargement of both atria, left pleural effusion, and signs of moderate pulmonary venous hypertension (Fig. 1). Two-dimensional echocardiography (2D ECHO) revealed a spectrum of severe valvular abnormalities, including rheumatic heart disease (RHD), mixed mitral valve disease comprising severe mitral stenosis (MS) with peak and mean gradient of 18 and 11 mm Hg (Fig. 2) with mitral valve area of 0.5 cm2, severe eccentric mitral regurgitation (MR) (Video 01), severe eccentric tricuspid regurgitation (TR) secondary to organic tricuspid valve involvement (Video 02), moderate pulmonary artery hypertension (PAH) (right ventricular systolic pressure = 42 mm Hg + right atrial pressure), mild aortic regurgitation (AR) without aortic stenosis (AS) due to aortic valve involvement (Video 03), giant left atrial enlargement (dimensions of 13 X 10 cm, calculated area 98 cm2) (Fig. 3), giant right atrial enlargement (dimensions of 10.5 X 8 cm, calculated area 65 cm2) (Fig. 4), yet with preserved biventricular function, mild pericardial effusion, and no evidence of left atrial clot formation. Cardiac computerised tomography revealed the grossly enlarged right atrium and left atrium without the evidence of thrombus formation in cardiac chambers (Fig. 5).

### Treatment

Her treatment protocol commenced with diuretics, beta-blockers, and anticoagulants, followed by a planned surgical intervention involving mitral valve replacement, tricuspid valve repair, and reduction of the left atrial volume. The surgical procedure successfully implemented a 29-mm mechanical mitral valve replacement (St Jude Medical bi-leaflet valve), tricuspid valve repair with annuloplasty ring, and left atrial volume reduction. The procedural bypass time was 210 min, and cross clamp time was 130 min.

### Follow-up

One month post-surgery, she exhibited remarkable symptomatic improvement, achieving New York Heart Association (NYHA) class I status, with normalised prosthetic valve function, minimal residual tricuspid regurgitation, absence of pulmonary artery hypertension, and restored biventricular function. She was continued on anticoagulants and penicillin prophylaxis.

## Discussion

Rheumatic heart disease (RHD) constitutes a significant burden on global health, affecting heart valves in a characteristic progression: primarily involving the mitral valve, followed by the aortic, tricuspid, and pulmonary valves. Multivalve involvement occurs in approximately one-third of RHD cases, with organic tricuspid valve pathology present in around 9.7% of instances [1]. Our index case presented a rare scenario with triple valve involvement (mitral, aortic, and tricuspid), with mixed mitral valve disease (MS and MR) being the prevailing lesions. This dual impact led to substantial left atrial enlargement, resulting in a giant left atrium (LA).

Giant LA, defined as a left atrial diameter exceeding 6.5 cm on M-mode echocardiography or radiographically touching the right lateral chest wall, is predominantly associated with rheumatic mitral valve disease [2, 3]. Remarkably, around 19% of patients undergoing mitral valve surgery present with a giant left atrium [4]. The pathophysiological consequences of MS and MR include significant volume and pressure overload on the left atrium, leading to various clinical sequelae such as atrial fibrillation, hoarseness of voice, Ortner's syndrome, and thromboembolism.

Conversely, giant right atrium is typically secondary to congenital abnormalities, idiopathic right atrial aneurysms, pulmonary embolism, or pulmonary hypertension, often linked to tricuspid valve abnormalities. However, in our case, the enlargement stemmed from both tricuspid valve regurgitation and pulmonary hypertension. Chronic pressure and volume overload resulting from mitral valvular dysfunction precipitated secondary pulmonary hypertension, further complicating the disease process. Instances of combined giant atria in a single patient are rare, underscoring the rarity and clinical significance of our observations.

In the existing literature, reports of either a giant left atrium or a giant right atrium in isolation are relatively common, while cases featuring a combination of both are exceedingly rare. Notably, the largest documented left atrium, measuring 22.3 × 19.2 × 20.1 cm, was observed in a 50-year-old woman, while the largest right atrium, measuring 18 × 15.3 × 16.3 cm, was reported in a 55-year-old woman with a disproportionately larger right atrium compared to the left [5, 6]. These cases underscore the extreme variations in atrial size seen in pathological conditions.

While similar cases have been reported, each presents unique characteristics that set it apart. For instance, a previously described case involving a 19-year-old female exhibited smaller dimensions compared to our case, likely due to differences in age and disease progression [7]. Furthermore, another reported instance in a 68-year-old woman occurred following mitral valve replacement, highlighting the distinct nature of our case, which involves the native valve and not a prosthetic replacement [8]. These variations emphasise the diverse spectrum of presentations and underscore the need for individualised approaches to diagnosis and management.

Early diagnosis and treatment drastically improve the morbidity and life expectancy in patients diagnosed with RHD. Recent identification of specific ARF biomarkers offers the opportunity to aid initial diagnosis, and portable echocardiography has the potential to detect undiagnosed RHD in high-risk areas. Using appropriate criteria given by World Heart Federation (WHF) help in identifying definite cases. Creating awareness and strict follow up will decrease the disease burden [9].

## Conclusions

Through our case study, we have learnt valuable lessons about the complexities of rheumatic heart disease and the importance of holistic management approaches. Despite facing significant socioeconomic challenges, our patient's journey highlights the potential for positive outcomes with diagnosis and comprehensive treatment strategies. By addressing the unique needs of individuals affected by RHD and advocating for improved access to healthcare resources, we can work towards reducing the global burden of this preventable and treatable condition.

### Learning objectives

- Understand the clinical manifestations and complications of rheumatic heart disease (RHD), including multivalve involvement and the development of giant atria.
- Recognise the importance of timely diagnosis and comprehensive management strategies in improving outcomes for patients with RHD.
- Appreciate the challenges and socioeconomic factors that contribute to the burden of RHD, particularly in underserved populations.
- Gain insights into the multidisciplinary approach to treating RHD, involving medical therapy, surgical intervention, and postoperative care.

### Supplementary Information

Additional file 1. Two-dimensional echocardiography—Apical four-chamber colour flow imaging showing severe mitral stenosis and severe eccentric mitral regurgitation

Additional file 2. Two-dimensional echocardiography—Apical four-chamber colour flow imaging showing severe eccentric tricuspid regurgitation

Additional file 3. Two-dimensional echocardiography—Apical five-chamber colour flow imaging showing mild aortic regurgitation

## Data Availability

Not applicable.

## Abbreviations

RHD: Rheumatic heart disease
NYHA: New York Heart Association
ECG: Electrocardiography
CXR: Chest X-ray
RA: Right atrium
LA: Left atrium
2D ECHO: Two-dimensional echocardiography
PAH: Pulmonary artery hypertension
TR: Tricuspid regurgitation
AS: Aortic stenosis
AR: Aortic regurgitation
